# ROVER variant caller: read-pair overlap considerate variant-calling software applied to PCR-based massively parallel sequencing datasets

**DOI:** 10.1186/1751-0473-9-3

**Published:** 2014-01-24

**Authors:** Bernard J Pope, Tú Nguyen-Dumont, Fleur Hammet, Daniel J Park

**Affiliations:** 1 Victorian Life Sciences Computation Initiative, 187 Grattan Street, Carlton, Melbourne, Victoria 3010, Australia; 2 Department of Computing and Information Systems, The University of Melbourne, Melbourne, Victoria 3010, Australia; 3 Genetic Epidemiology Laboratory, Department of Pathology, Medical Building, The University of Melbourne, Melbourne, Victoria 3010, Australia

**Keywords:** PCR-MPS, Hi-Plex, Targeted sequencing, Massively parallel sequencing, Variant calling, ROVER variant caller

## Abstract

**Background:**

We recently described Hi-Plex, a highly multiplexed PCR-based target-enrichment system for massively parallel sequencing (MPS), which allows the uniform definition of library size so that subsequent paired-end sequencing can achieve complete overlap of read pairs. Variant calling from Hi-Plex-derived datasets can thus rely on the identification of variants appearing in both reads of read-pairs, permitting stringent filtering of sequencing chemistry-induced errors. These principles underly ROVER software (derived from Read Overlap PCR-MPS variant caller), which we have recently used to report the screening for genetic mutations in the breast cancer predisposition gene *PALB2*. Here, we describe the algorithms underlying ROVER and its usage.

**Results:**

ROVER enables users to quickly and accurately identify genetic variants from PCR-targeted, overlapping paired-end MPS datasets. The open-source availability of the software and threshold tailorability enables broad access for a range of PCR-MPS users.

**Methods:**

ROVER is implemented in Python and runs on all popular POSIX-like operating systems (Linux, OS X). The software accepts a tab-delimited text file listing the coordinates of the target-specific primers used for targeted enrichment based on a specified genome-build. It also accepts aligned sequence files resulting from mapping to the same genome-build. ROVER identifies the amplicon a given read-pair represents and removes the primer sequences by using the mapping co-ordinates and primer co-ordinates. It considers overlapping read-pairs with respect to primer-intervening sequence. Only when a variant is observed in both reads of a read-pair does the signal contribute to a tally of read-pairs containing or not containing the variant. A user-defined threshold informs the minimum number of, and proportion of, read-pairs a variant must be observed in for a ‘call’ to be made. ROVER also reports the depth of coverage across amplicons to facilitate the identification of any regions that may require further screening.

**Conclusions:**

ROVER can facilitate rapid and accurate genetic variant calling for a broad range of PCR-MPS users.

## Background

Targeted massively parallel sequencing (MPS) offers significantly increased efficiencies compared with prior technologies such as Sanger sequencing and High-Resolution Melting curve analysis (HRM) for diverse genetic analysis applications [1,2]. Such applications include disease gene discovery and mutation screening, and clinical diagnostic gene-panel testing [3,4]. Among PCR-based approaches for MPS, we recently described a single-reaction, highly-multiplexed PCR-MPS system called Hi-Plex, designed to enable rapid and cost-effective targeted sequencing [5,6]. Among the advantages offered by this system is the ability to define the target library size so that contemporary paired-end MPS sequencing protocols can readily result in both members of a read-pair completely covering the primer-intervening sequence of the relevant amplicon. Consequently, high-stringency filtering of sequencing chemistry errors is possible by comparing both reads of a pair over the entire read-pair length. Conceptually, this approach to PCR-MPS data handling is attractive irrespective of the upstream molecular biology. Low-level residual system noise resulting from PCR polymerase error could readily be accounted for by classical duplicate testing of specimens. This will be very useful in cases where genetic variants are present in a relatively small fraction of the whole sample, as is often the case for somatic mutations in tumour biopsies, for example.

Contemporary approaches to handling PCR-MPS data include the application of tools to ‘trim’ primer or adapter sequences or ‘merge’ read-pairs to yield ‘consensus’ read representations. For example, Trimmomatic can be used to detect and remove adapter sequences or other specified sequences at the beginning and end of a read, but discards the complementary read of the read-pair [7]. Identification of sequence to be trimmed based on anticipated adapter and/or other oligonucleotide sequences can be problematic because mismatches occur commonly due to oligonucleotide synthesis and sequencing chemistry errors. SeqPrep merges paired-end Illumina sequencing chemistry reads that are overlapping into a single longer read. Sequence identity thresholds are used to determine whether adapter sequences are present and whether read pairs overlap [8]. Trimming of reads is supported and in the context of reads-overlap having been identified, a single ‘consensus’ representation of the read-pair is produced. The accuracy of this approach depends on the relative quality measures attributed to the two reads of a pair. Incorrect consensus ‘reads’ will be derived in situations where sequencing errors are attributed with relatively high quality scores and/or the corresponding positions of the non-erroneous reads receive relatively low quality scores.

In settings where accuracy is a high priority, we reasoned that an approach that requires agreement between both reads of a read-pair for a given position before the read-pair is allowed to contribute to variant-calling, would be desirable. Furthermore, by using co-ordinates information relating to primers used in PCR-MPS applications, read-trimming should theoretically be possible without the confounding influences of errors introduced during primer synthesis or sequencing. To realise these design features, we developed ROVER software which enables highly-accurate detection of genetic variants in PCR-MPS datasets by applying filtering based on completely overlapping reads in read-pairs. Amplicon library insert size should be shorter than the length of a sequencing read for compatibility with this software. Previously, we reported the use of ROVER in the context of screening for genetic mutations in the breast cancer predisposition gene, *PALB2* [GenBank reference sequence NM_024675; MIM#610355] [9]. Here, we describe its mode of action and usage.

## Implementation

ROVER is implemented in Python 2.7 and is intended to be used as a command-line application. Its two key inputs are 1) a tab-delimited file of DNA co-ordinates specifying the locations of target amplicon regions, relative to a reference sequence, in standard BED format [10]; 2) one or more files containing reads aligned to the reference sequence in the standard BAM format [11]. Its main output is a file containing a list of variants called for each input BAM file in tab-delimited format compatible with widely-used variant annotation tools such as ANNOVAR [12]. It also accepts two threshold arguments which are used to filter the list of resulting variants as described below. The types of variants considered by ROVER are single nucleotide variants (SNVs) and small insertions and deletions (indels).

**Figure 1:**
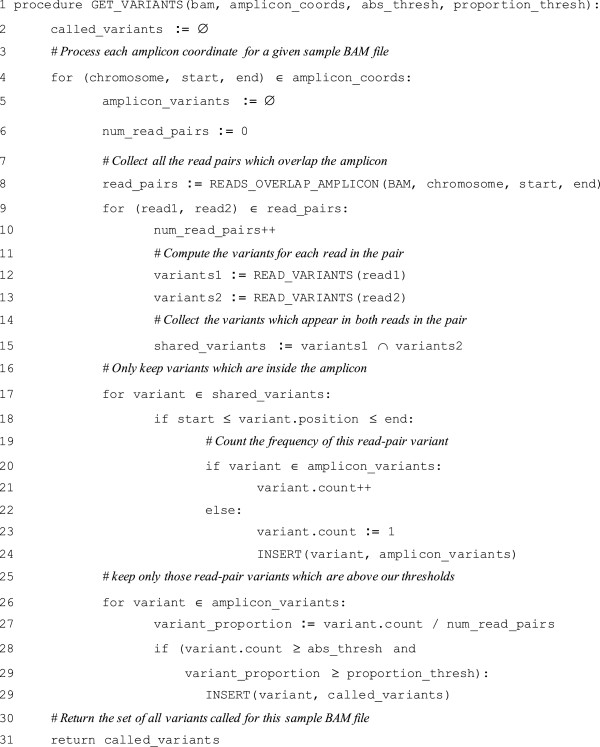
Pseudocode illustrating the main variant calling algorithm employed by ROVER.

The pseudocode in Figure 1 illustrates the algorithm employed by ROVER to compute variants for a single input BAM file. The algorithm is applied to each input BAM file and the results are accumulated and then saved to the output file.

The algorithm is realised by the GET_VARIANTS function which takes four parameters: 1) the input BAM file; 2) the list of amplicon co-ordinates; 3) the absolute threshold for the number of read-pairs required for a variant to be kept; 4) the proportion threshold for the number of read-pairs required for the variant to be kept. It returns as its result a set of variants which are called for this input BAM file.

**Figure 2:**
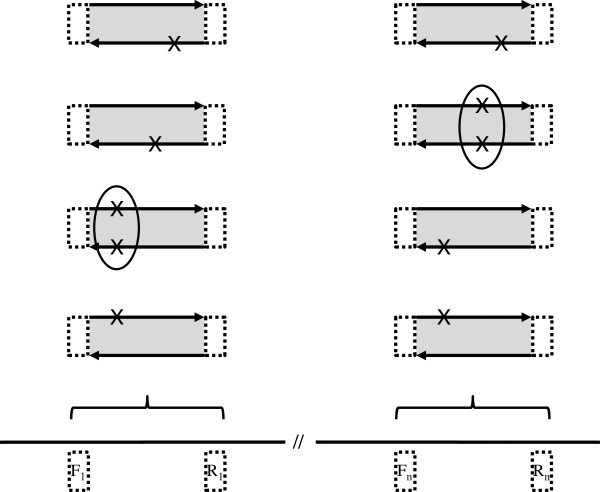
**Illustration of the ROVER principles.** The lower panel represents contiguous sections of a reference sequence (e.g. a specific human genome build). Numbered ‘F’ and ‘R’ pairs denote primer co-ordinates used in conjunction with mapped read starting co-ordinates to guide clipping (dotted boxes). Shaded boxes represent regions under analysis. Arrows represent reads of read-pairs. ‘X’s denote positions different from the reference sequence. Only when both reads of a read-pair concur can the read-pair contribute to genetic variant calling for a given position (circled). This contribution to a read-pair pile-up allows variant calling using thresholds for minimum number of read-pairs and the proportion of read-pairs containing the variant.

Each amplicon is processed separately by the loop from line 4 to line 29. All the read-pairs which overlap the amplicon are retrieved from the input BAM file (line 8). In practice, the fraction that a read must overlap an amplicon can be tailored by a command-line argument, defaulting to 0.9. It is important to note that we only consider reads which are part of a mapped pair. For each pair of reads, we compute the set of variants which appear in *both* reads (lines 9 to 15). We then discard any variants which appear on both reads but are not within the bounds of the amplicon (lines 17 to 24) (see Figure 2). For each variant which appears within the amplicon bounds, we also count the number of times that variant is seen in all the pairs which overlap the amplicon (lines 21 and 23). We then discard any variants which do not meet the threshold requirements (lines 26 to 29). The first threshold is the absolute count of read-pairs for a variant in a given amplicon. We must see a variant in at least this many read-pairs overlapping the amplicon for it to be kept in the output. The second threshold is the proportion of read-pairs containing a variant in a given amplicon. We must see a variant in at least this proportion of all the read-pairs overlapping the amplicon for it to be kept in the output. Variants which pass both of the thresholds are added to the overall set of called variants (line 29). After processing all the amplicons, the set of all called variants is returned as the result (line 31). An optional input argument allows the user to dictate that only when base changes or insertions are associated with quality scores in both reads above a selectable threshold, will a read-pair be considered in variant calling.

To compute the variants in a particular read we compare the aligned nucleotide bases of the read with the reference sequence. The so-called CIGAR string of mapped reads, given in the BAM file for each read, is used to relate the bases of the read to the positions of the reference sequence. Additionally, the bases from the reference sequence are deduced from the MD field of the BAM file. This allows ROVER to recover the reference sequence just from the BAM file without needing the original reference file to be specified. ROVER uses the PySam library for manipulating BAM files [13].

## Results and discussion

ROVER was developed and released as open source software. ROVER performs trimming of primer sequences based on a co-ordinates file, avoiding problems associated with sequence matching in the context of errors introduced during primer synthesis, polymerase activity and sequence detection. The software considers overlapping read-pairs (as far as the start of distal primer-corresponding sequence for each read of a read-pair) and only allows read-pair concordant variants to be used for variant calling. ROVER allows thresholds for read-pair depth and read-pair proportion to be modified. The software also produces a report of mapped read-pair numbers for each amplicon, allowing the user to monitor coverage.

Using a BAM file consisting of 52,592 reads of 150 bases, derived from a Hi-Plex experiment targeting 60 amplicons,[9], ROVER took 7 seconds and required 14 MB of memory to call the variants and compute the depth of coverage across amplicons, on an Apple Mac Pro with two 2.66 GHz 6-Core processor and 64 GB RAM running OS 10.8.4. In instances where multiple BAM files are analysed, the software can be run as follows (italics indicate example parameter values):

rover --primers *hiplex_primers.txt* --log *logfile.txt* --out *variant_calls.tsv*

--proportionthresh *0.15* --absthresh *2* --qualthresh *20* --coverdir *coverage_dir*

sampleX.bam sampleY.bam sampleZ.bam

In the era of MPS-based high-throughput sequencing both in research and clinical settings, one of the biggest challenges is the generation of false-positive signals. The requirement by ROVER for read-pair concordance essentially restricts chemistry-induced error to polymerase misincorporation during PCR. Using germline DNA as input material, this source of error can readily be filtered out by application of depth and proportion of read-pairs thresholds, since many copies of PCR template (typically hundreds or thousands) are generally present at the outset of a PCR reaction and it is highly unlikely for a significant proportion of early amplification events to result in identical polymerase errors. In more challenging settings, for example the identification of rare somatic genetic variants in tumour biopsies, duplicate testing in conjunction with ROVER will afford highly accurate variant calling. Other sources of error, such as alignment errors arising from segmental duplication, can be accounted for by comparison across specimens treated using the same chemistry and bioinformatic analysis pipeline, using principles we have described previously [14].

As reported in [9], application of ROVER to a high-throughput (96-well format) Hi-Plex-based dataset enabled rapid and accurate identification of genetic variants in the coding regions of *PALB2*. In this blinded study, 99.93% (5,696/5,700) of amplicons were represented at ≥10× coverage, across samples. We accurately detected all 56 variant calls identified through previous mutation screening and genotyping. Heterozygous variants were observed in 37.23% (35/94) to 62.33% (513/823) of read-pairs (median = 51.23%). No false positive calls were assigned.

## Conclusions

ROVER is a freely available open-source tool that enables accurate genetic variant calling from paired-end PCR-MPS datasets in which reads completely overlap for the regions under analysis.

## Availability and requirements

Project name: ROVER

Project home page: https://github.com/bjpop/rover

Operating systems: POSIX-like operating systems (OS X, Linux)

Programming language: Python

Other requirements: PySam library

License: BSD

Any restrictions to use by non-academic: None

## Abbreviations

MPS: Massively parallel sequencing; derived from Read Overlap PCR-MPS variant caller: ROVER software; HRM: High Resolution Melting-curve analysis; IGV: Integrative genome viewer.

## Competing interests

The authors declare that they have no competing interests.

## Authors’ contributions

BJP and DJP conceived the design of the software. BJP performed the coding. FH and TN-D conducted testing of the software. BJP, TN-D, FH and DJP contributed to the writing of the manuscript. All authors read and approved the final manuscript.

## References

[B1] Mardis ER The impact of next-generation sequencing technology on genetics Trends Genet 2008 24 133 141 10.1016/j.tig.2007.12.007 18262675 PMC2680276

[B2] Moorthie S Mattocks CJ Wright CF Review of massively parallel DNA sequencing technologies The HUGO Journal 2011 5 1 12 23205160 10.1007/s11568-011-9156-3 PMC3238019

[B3] Morgan JE Carr IM Sheridan E Chu CE Hayward B Camm N Lindsay HA Mattocks CJ Markham AF Bonthron DT Taylor GR Genetic diagnosis of familial breast cancer using clonal sequencing Hum Mutat 2010 31 484 491 10.1002/humu.21216 20127978

[B4] Meldrum C Doyle MA Tothill RW Next-generation sequencing for cancer diagnostics: a practical perspective Clin Biochem Rev 2011 32 177 195 22147957 PMC3219767

[B5] Nguyen-Dumont T Pope BJ Hammet F Southey MC Park DJ A high-plex PCR approach for massively parallel sequencing Biotechniques 2013 55 69 74 23931594 10.2144/000114052

[B6] Nguyen-Dumont T Pope BJ Hammet F Mahmoodi M Tsimiklis H Southey MC Park DJ Cross-platform compatibility of Hi-Plex, a streamlined approach for targeted massively parallel sequencing Anal Biochem 2013 442 2 127 129 10.1016/j.ab.2013.07.046 23933242 PMC3839948

[B7] Trimmomatic: a flexible read trimming tool for Illumina NGS data http://www.usadellab.org/cms/index.php?page=trimmomatic

[B8] SeqPrep https://github.com/jstjohn/SeqPrep

[B9] Nguyen-Dumont T Teo ZL Pope BJ Hammet F Mahmoodi M Tsimiklis H Sabbaghian N Tischkowitz M Foulkes WD Giles GG Hi-Plex for high-throughput mutation screening: application to the breast cancer susceptibility gene *PALB2* BMC Med Genomics 2013 6 48 10.1186/1755-8794-6-48 24206657 PMC3829211

[B10] Quinlan AR Hall IM BEDTools: a flexible suite of utilities for comparing genomic features Bioinformatics 2010 26 841 842 10.1093/bioinformatics/btq033 20110278 PMC2832824

[B11] Li H Handsaker B Wysoker A Fennell T Ruan J Homer N Marth G Abecasis G Durbin R 1000 Genome Project Data Processing Subgroup The Sequence Alignment/Map format and SAMtools Bioinformatics 2009 25 2078 2079 10.1093/bioinformatics/btp352 19505943 PMC2723002

[B12] Wang K Li M Hakonarson H ANNOVAR: functional annotation of genetic variants from high-throughput sequencing data Nucleic Acids Res 2010 38 e164 10.1093/nar/gkq603 20601685 PMC2938201

[B13] PySam: Python interface for the SAM/BAM sequence alignment and mapping format http://code.google.com/p/pysam/

[B14] Pope BJ Nguyen-Dumont T Odefrey F Hammet F Bell R Tao K Tavtigian SV Goldgar DE Lonie A Southey MC Park DJ FAVR (Filtering and Annotation of Variants that are Rare): methods to facilitate the analysis of rare germline genetic variants from massively parallel sequencing datasets BMC Bioinforma 2013 14 65 10.1186/1471-2105-14-65 PMC3599469 23441864

